# The relation between proteinuria and the severity of COVID-19

**DOI:** 10.1007/s10157-023-02428-9

**Published:** 2023-11-14

**Authors:** Akira Fukui, Kohei Takeshita, Akio Nakashima, Yukio Maruyama, Nobuo Tsuboi, Tokio Hoshina, Takashi Yokoo

**Affiliations:** 1 Division of Nephrology and Hypertension, Department of Internal Medicine, Jikei University School of Medicine, 3-25-8 Nishi-Shimbashi Minato-Ku, Tokyo, 105-8471 Japan; 2 Department of Innovation for Medical Information Technology, Jikei University School of Medicine, Tokyo, Japan; 3 Department of Infectious Diseases and Infection Control, Jikei University School of Medicine, Tokyo, Japan

**Keywords:** Chronic kidney disease (CKD), Proteinuria, Estimated glomerular filtration rate (eGFR), COVID-19 severity, Severe acute respiratory syndrome coronavirus 2 (SARS-CoV-2)

## Abstract

**Background:**

The association between proteinuria, which is also an indicator of chronic kidney disease (CKD), and coronavirus disease 2019 (COVID-19) severity is unclear.

**Methods:**

We selected 342 hospitalized patients with COVID-19 diagnosed via polymerase chain reaction testing between February 2020 and October 2022 and who had at least one urinalysis 14–365 days before admission.

**Results:**

Proteinuria before admission was associated neither with oxygen administration nor developing pneumonia in multivariate analysis (odds ratio [OR] 1.03; 95% confidence interval (CI) 0.44–2.40, p = 0.95 and OR 1.01; 95% CI 0.47–2.17, p = 0.98, respectively). Proteinuria on admission was associated both with oxygen administration and developing pneumonia in multivariate analysis (OR 3.29; 95% CI 1.37–7.88, p < 0.01 and OR 3.81; 95% CI 1.68–8.62, p < 0.01, respectively). The percentage of patients with proteinuria on admission was significantly higher than those before admission (37.4% vs. 17.8%; p < 0.01). In the subgroup analysis, proteinuria on admission among patients with eGFR ≥ 60 mL/min/1.73 m^2^ was associated with both oxygen administration and developing pneumonia (OR 4.86; 95% CI 1.22–19.38, p = 0.03, OR 3.65; 95% CI 1.06–12.58, p = 0.04, respectively). In contrast, proteinuria on admission among patients with eGFR < 60 mL/min/1.73 m^2^ was associated with developing pneumonia (OR 6.45; 95%CI 1.78–23.35, p = 0.01), not with oxygen administration (OR 3.28; 95% CI 0.92–11.72, p = 0.07).

**Conclusions:**

Although underlying proteinuria before admission was not associated with COVID-19 severity, proteinuria on admission was associated with oxygen demand and developing pneumonia.

**Supplementary Information:**

The online version contains supplementary material available at 10.1007/s10157-023-02428-9.

## Introduction

Beginning in early 2023, the severe acute respiratory syndrome coronavirus 2 (SARS-CoV-2) infection-related pandemic of coronavirus disease 2019 (COVID-19) will continue to harm people's health and place a strain on medical services worldwide. Chronic kidney disease (CKD) is a key risk factor for the severity of COVID-19 and one of the main targets of SARS-CoV-2 [1–4]. Proteinuria and acute kidney injury (AKI) are also known to be frequently observed shortly after infection with SARS-CoV-2 by its direct and indirect effects [5–10]. Hence, values of renal parameters before admission may be necessary to evaluate the risk of underlying CKD, but there are not many such studies, and the reduced estimated glomerular filtration rate (eGFR) rather than proteinuria is defined as CKD [11]. However, proteinuria is an established indicator of CKD and is reported to be associated with vascular endothelial dysfunction, inflammation, aging, diabetes mellitus, obesity, and heart failure, all of which are also linked to COVID-19 severity [12–20].

The objective of this study is to examine the relationship between non-dialysis-dependent CKD, particularly focusing on proteinuria, and COVID-19 severity. Moreover, we compared the values of renal parameters on admission with those before admission.

## Materials and methods

### Patients group and study structure

**Fig. 1:**
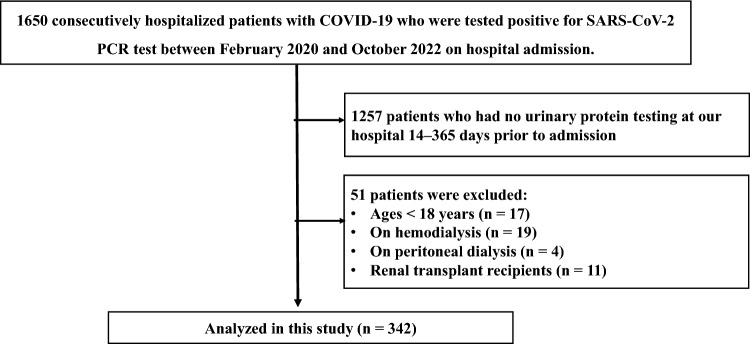
Flowchart. We used the registry of consecutive 1650 hospitalized patients with COVID-19 who were tested positive for the SARS-CoV-2 PCR test between February 9, 2020, and October 30, 2022, on hospital admission. Of the 1650 patients, 393 patients who had at least one urinary protein test at our hospital 14–365 days before admission were extracted using the data warehouse CLISTA! 3.5 (Medical Engineering Institute, Inc., Tsu, Japan) [21]. Ages < 18 years (n = 17) and patients receiving hemodialysis (n = 19), peritoneal dialysis (n = 4), and renal transplant (n = 11) were excluded. Thus, the final analysis included 342 patients

The data of the study were from Jikei University Hospital in Tokyo, Japan, which employs computerized medical records. Between February 9, 2020, and October 30, 2022, a total of 1650 consecutive patients with COVID-19 who were admitted also tested positive for SARS-CoV-2 via polymerase chain reaction (PCR). The registry includes the data on age, sex, smoking, comorbid diseases, and medical information on COVID-19 (pneumonia findings diagnosed via CT scan or chest X-ray during hospitalization, the requirement of oxygen administration, the requirement of mechanical ventilation, and in-hospital mortality). The definition of cardiovascular disease and the term “lung disease” refer to Supplementary Methods. Of the 1650 patients, 393 patients who had at least one urinary dipstick protein testing at our hospital 14–365 days before admission were obtained using the data warehouse CLISTA! 3.5 (Medical Engineering Institute, Inc., Tsu, Japan) [21]. Then, ages < 18 years (n = 17) and patients on hemodialysis (n = 19), peritoneal dialysis (n = 4), and renal transplant recipients (n = 11) were excluded. Thus, the final analysis included 342 patients (Fig. 1). We recorded values of their biochemical measurements, such as urinary protein, urinary occult blood, serum creatinine, and eGFR, and body weight, height, and body mass index (BMI) from electronic medical records before admission, those on admission soon after SARS-CoV-2 diagnosis and finally those at the time of discharge. We also obtained regarding body temperature and inflammation-related parameters, such as CRP, leukocyte and lymphocyte levels on admission. We employed the most recent values from at least 14 days before admission, with due consideration of the incubation period of SARS-COV-2 [22]. Conversely, the values on admission were obtained from the first measurement after admission. The following eGFR equation, created for the Japanese population, was used to determine eGFR calculated as follows: 194 × (serum creatinine (mg/dL))^−1.094^ × (age (years old))^−0.287^ (× 0.739, for female). A urine dipstick test was performed with urine test strips (UrifletS-9UB, ARKRAY, Kyoto, Japan) and a fully automated urine analyzer (AUTION MAX AX-4061, ARKRAY, Kyoto, Japan) during the whole study period. Positive proteinuria was defined as dipstick proteinuria ≥ 1 + (≥ 30 mg/dL) and reduced eGFR was defined as eGFR < 60 mL/min/1.73 m^2^ [23].

### Outcomes

The outcome was in-hospital mortality, the requirement of mechanical ventilators, the requirement of oxygen administration, and developing pneumonia during hospitalization.

### Statistical analysis

Medians are used to describe non-normally distributed data (interquartile ranges). Data that are normally dispersed are presented as mean ± standard deviation. Numbers and percentages are used to describe binary data. Student’s *t* test or the Wilcoxon rank sum test, as applicable, was used to analyze continuous variables, whereas the *χ*^2^ test was used to analyze nominal variables. Multiple logistic regression analysis was performed for oxygen administration or developing pneumonia. The explanatory variables are as follows: age, sex, BMI, smokers, diabetes mellitus, a history of cardiovascular disease or lung disease, urine protein, and underlying eGFR. In subgroup analysis, we evaluated the association between proteinuria and outcome among patients with eGFR < 60 mL/min/1.73 m^2^ and those with eGFR ≥ 60 mL/min/1.73 m^2^. Statistical significance was defined as a p < 0.05. STATA version 17.0 (STATA Corp., College Station, TX, USA) was used to conduct all statistical tests.

## Results

### Population characteristics of the research

Table 1 displays the characteristics of the research participants (n = 342). The mean age on admission was 63 ± 17 years, and 219 participants (64.0%) were men. Among the 342 patients, 122 were diagnosed with diabetes, 113 with hypertension, and 5 with IgA nephropathy. The renal parameters before admission indicated that 17.8% of participants had positive proteinuria and 34.7% had reduced eGFR. Twenty-six among the 58 cases with positive proteinuria were hospitalized after January 2022, a period when the Omicron strain was known to cause COVID-19 in Japan. Table 2 shows the comparison of parameters for each outcome. Regarding the individual components, there were 14 (4.1%) in-hospital mortality, 19 (5.7%) patients required mechanical ventilators, 93 (27.7%) required oxygen administration, and 148 (45.7%) patients had pneumonia findings among the entire study population. The data for 18 cases of progression to pneumonia, 6 cases of oxygenation, and 7 cases of ventilator management are missing.

**Table 1:** Baseline characteristics

Variables	Values
Number	342
Age [years]	63 ± 17
Male (%)	219 (64.0%)
Height [cm]	165 ± 10
BW [kg]	64.2 ± 15.6
BMI [kg/m^2^]	23.5 ± 4.6
Comorbid disease	
Diabetes [%]	122 (36.4%)
Hypertension [%]	113 (33.0%)
Cardiovascular disease [%]	38 (11.3%)
Lung disease [%]	79 (23.6%)
IgA nephropathy [%]	5 (1.5%)
SLE [%]	4 (1.2%)
Membranous nephropathy [%]	3 (0.9%)
Minimal change nephrotic syndrome [%]	2 (0.6%)
Smoking status	
Smoker [%]	133 (57.8%)
Never smoker [%]	97 (42.2%)
Renal parameters before admission	
Cr [mg/dL]	0.83 (0.67–1.04)
eGFR [mL/min/1.73m^2^]	68.9 ± 27.3
eGFR < 60 mL/min/1.73m^2^ [%]	118 (34.7%)
Proteinuria ≥ 1 + [%]	58 (17.8%)
Renal parameters on admission	
Cr [mg/dL]	0.85 (0.66–1.09)
eGFR [mL/min/1.73m^2^]	66.5 ± 26.9
eGFR < 60 mL/min/1.73m^2^ [%]	129 (39.0%)
Proteinuria ≥ 1 + [%]	76 (37.4%)

Means ± SD or medians and interquartile range (IQR)

*BW*, body weight; *BMI*, body mass index; *eGFR*, estimated glomerular filtration rate

**Table 2:** Univariate analyzes for comparisons of baseline characteristics

No. of patients tested for proteinuria before admission	Developing pneumonia			Oxygen administration			Mechanical ventilators			In-hospital mortality		
	With	Without	P	With	Without	P	With	Without	P	Death	Surviver	P
	n = 148	n = 176		n = 93	n = 243		n = 19	n = 316		n = 14	n = 328	
Age [year]	65 ± 15	63 ± 18	0.17	70 ± 12	61 ± 18	< 0.01	71 ± 9	63 ± 17	0.048	74 ± 9	63 ± 17	0.02
Male gender	104 (70.3%)	107 (60.8%)	0.08	69 (74.2%)	149 (61.3%)	0.02	17 (89.5%)	200 (63.3%)	0.02	10 (71.4%)	209 (63.7%)	0.78
BMI [kg/m^2^]	24.0 ± 4.9	22.9 ± 4.2	0.04	24.1 ± 4.7	23.2 ± 4.5	0.11	24.6 ± 4.6	23.4 ± 4.6	0.25	21.8 ± 3.8	23.6 ± 4.6	0.16
Diabetes	63 (42.6%)	56 (32.0%)	0.06	45 (48.4%)	77 (32.1%)	< 0.01	9 (47.4%)	112 (35.8%)	0.33	8 (57.1%)	114 (35.5%)	0.15
Cardiovascular disease	20 (13.5%)	18 (10.3%)	0.39	9 (9.7%)	29 (12.1%)	0.70	2 (10.5%)	35 (11.2%)	1.00	3 (21.4%)	35 (10.9%)	0.20
Lung disease	38 (25.7%)	37 (21.1%)	0.36	32 (34.4%)	46 (19.2%)	< 0.01	6 (31.6%)	71 (22.7%)	0.40	6 (42.9%)	73 (22.7%)	0.11
Smoking	57 (60.6%)	71 (55.0%)	0.41	46 (71.9%)	86 (52.1%)	< 0.01	9 (75.0%)	123 (56.9%)	0.25	9 (75.0%)	124 (56.9%)	0.25
*Renal parameters before admission*												
eGFR [mL/min/1.73m^2^]	64.8 ± 22.9	70.4 ± 27.9	0.06	61.4 ± 21.3	70.9 ± 28.0	< 0.01	61.3 ± 14.2	68.7 ± 27.2	0.24	54.1 ± 21.7	69.5 ± 27.3	0.04
eGFR < 60 mL/min/1.73m^2^ [%]	60 (40.5%)	55 (31.6%)	0.10	42 (45.2%)	76 (31.5%)	0.01	9 (47.4%)	109 (34.7%)	0.32	8 (57.1%)	110 (33.7%)	0.09
Proteinuria ≥ 1 + [%]	25 (17.5%)	31 (18.8%)	0.88	16 (18.2%)	42 (18.1%)	1.00	1 (5.3%)	57 (19.0%)	0.22	3 (21.4%)	55 (17.6%)	0.72
*Renal parameters on admission*												
eGFR [mL/min/1.73m^2^]	62.2 ± 26.4	69.2 ± 26.9	0.02	58.8 ± 23.8	68.7 ± 27.1	< 0.01	57.3 ± 17.7	66.4 ± 27.0	0.16	49.3 ± 20.1	67.2 ± 26.9	0.02
eGFR < 60 mL/min/1.73m^2^ [%]	66 (44.9%)	61 (35.9%)	0.11	47 (51.7%)	82 (34.9%)	< 0.01	11 (61.1%)	118 (38.4%)	0.08	10 (76.9%)	119 (37.4%)	< 0.01
Proteinuria ≥ 1 + [%]	50 (48.1%)	24 (25.5%)	< 0.01	33 (54.1%)	43 (37.8%)	< 0.01	3 (23.1%)	73 (39.0%)	0.38	5 (55.6%)	71 (36.6%)	0.30

*BMI*, body mass index; *eGFR*, estimated glomerular filtration rate

### Factors associated with each outcome

Univariate analysis of the association between each outcome and renal parameters was performed. Proteinuria before admission was not associated with any outcome, whereas proteinuria on admission was associated with oxygen administration and developing pneumonia (Table 2). Reduced eGFR before admission was associated only with oxygen administration, whereas reduced eGFR on admission was associated with oxygen administration and in-hospital mortality (Table 2). Since the in-hospital mortality rate and the rate of the use of mechanical ventilators were low in our study population, multivariate logistic regression analysis was performed for oxygen administration and developing pneumonia.

In the analysis using renal parameters before admission, proteinuria before admission was associated neither with oxygen administration nor developing pneumonia in multivariate analysis (odds ratio [OR] 1.03; 95% confidence interval (CI) 0.44–2.40, p = 0.95, and OR 1.01; 95% CI 0.47–2.17, p = 0.98, respectively) (Tables 3 and 4). Reduced eGFR before admission was associated neither with oxygen administration nor developing pneumonia in multivariate analysis (OR 1.05; 95% CI 0.52–2.12, p = 0.90 and OR 1.26; 95% CI 0.66–2.42, p = 0.49, respectively) (Tables 3 and 4). In multivariate analysis, older age was also associated with a larger ratio of oxygen administration (OR 1.04; 95% CI 1.01–1.07, p 0.01) (Tables 3 and 4).

**Table 3:** Factors contributing to the requirement of oxygen administration

Variable	Using renal parameters before admission			Using renal parameters on admission		
	Odds ratio	95% CI	p value	Odds ratio	95% CI	p value
Age [years]	1.04	1.01–1.07	< 0.01	1.04	1.01–1.08	0.02
Male gender	1.73	0.72–4.14	0.22	3.95	1.19–13.07	0.03
BMI [kg/m^2^]	1.05	0.97–1.13	0.21	1.09	0.99–1.19	0.08
Smokers	1.42	0.64–3.13	0.39	0.62	0.23–1.72	0.36
Diabetes	1.52	0.78–2.97	0.22	1.20	0.51–2.79	0.68
Cardiovascular disease	0.66	0.24–1.86	0.43	0.51	0.16–1.66	0.27
Lung disease	1.65	0.80–3.39	0.17	2.03	0.79–5.25	0.14
Proteinuria ≥ 1 +	1.03	0.44–2.40	0.95	3.29	1.37–7.88	< 0.01
eGFR < 60 mL/min/1.73m^2^	1.05	0.52–2.12	0.90	0.84	0.33–2.14	0.71

*CI*, confidence interval; *BMI*, body mass index; *eGFR*, estimated glomerular filtration rate

**Table 4:** Factors contributing to the developing pneumonia

Variable	Using renal parameters before admission			Using renal parameters on admission		
	Odds ratio	95% CI	p value	Odds ratio	95% CI	p value
Age [years]	1.01	0.99–1.03	0.32	1.01	0.98–1.03	0.70
Male gender	1.07	0.52–2.18	0.86	1.07	0.43–2.67	0.89
BMI [kg/m^2^]	1.03	0.96–1.09	0.44	1.07	0.99–1.16	0.10
Smokers	1.10	0.56–2.17	0.78	1.03	0.43–2.48	0.94
Diabetes	1.04	0.57–1.92	0.90	0.96	0.44–2.08	0.92
Cardiovascular disease	2.62	0.97–7.09	0.06	1.82	0.59–5.57	0.29
Lung disease	1.20	0.61–2.35	0.60	2.12	0.86–5.21	0.10
Proteinuria ≥ 1 +	1.01	0.47–2.17	0.98	3.81	1.68–8.62	< 0.01
eGFR < 60 mL/min/1.73m^2^	1.26	0.66–2.42	0.49	1.30	0.56–3.00	0.55

In the analysis using renal parameters on admission, proteinuria on admission was associated both with oxygen administration and developing pneumonia in multivariate analysis (OR 3.29; 95% CI 1.37–7.88, p < 0.01 and OR 3.81; 95% CI 1.68–8.62, p < 0.01, respectively) (Tables 3 and 4). Reduced eGFR on admission was associated neither with oxygen administration nor developing pneumonia in multivariate analysis (OR 0.84; 95% CI 0.33–2.14, p = 0.71 and OR 1.30; 95% CI 0.56–3.00, p = 0.55, respectively) (Tables 3 and 4). Additionally, in a multivariate analysis, a greater ratio of oxygen administration was associated with older age and male gender (OR 1.04; 95% CI 1.01–1.08, p = 0.02 and OR 3.95; 95% CI 1.19–13.07, p = 0.03, respectively) (Tables 3 and 4). We also analyzed the association between proteinuria and various inflammation-related parameters (Supplementary Table 1). Among these parameters, CRP (negative proteinuria: 1.44 (0.53 to 4.35) vs positive proteinuria: 3.75 (1.51 to 9.88), p < 0.01) and hematuria (negative proteinuria: 29/124 (23.4%) vs positive proteinuria: 39/74 (52.7%), p < 0.01) were associated with proteinuria on admission. We also analyzed the association between these parameters and outcomes (Supplementary Table 2).

### Comparison of the value of renal parameters on admission with those before admission or at the time of discharge

**Fig. 2:**
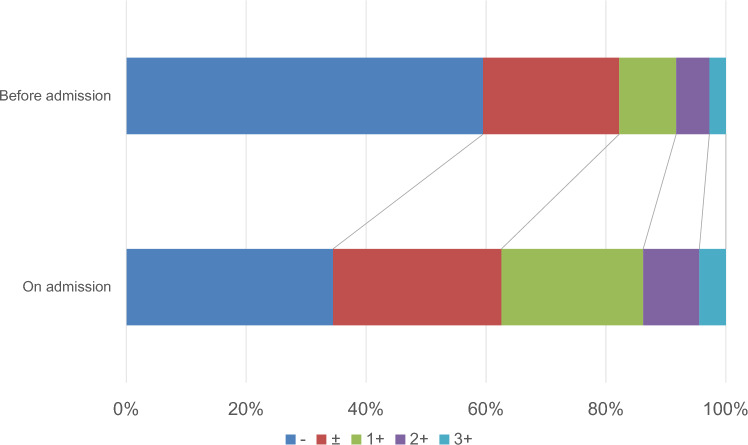
Changes in proteinuria. Uric protein measured via the dipstick test on admission was significantly more severe than those before admission (chi-square, p < 0.01)

**Fig. 3:**
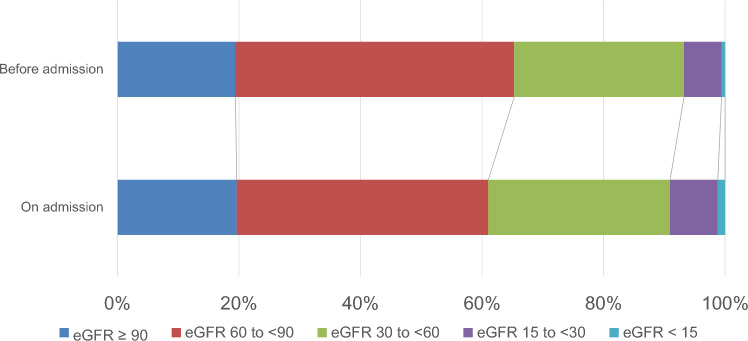
Changes in eGFR. EGFR on admission was significantly lower than those before admission (chi-square, p < 0.01)

Uric protein measured by the dipstick test on admission was significantly more severe than those before admission (chi-square, p < 0.01, Fig. 2), and the percentage of patients with proteinuria on admission was significantly higher than those before admission (37.4% vs. 17.8%; chi-square, p < 0.01, Table 1). Similarly, eGFR on admission was significantly lower than those before admission (chi-square, p < 0.01, Fig. 3), and the proportions of individuals whose eGFR is under 60 mL/min/1.73 m^2^ on admission were significantly higher than those before admission (39.0% vs. 34.7%; chi-square, p < 0.01, Table 1). We also analyzed the association between changes in proteinuria and patient outcomes (Supplementary Table 3). The novel proteinuria was observed to be associated with oxygen administration and pneumonia. However, other groups were difficult to assess because of their low frequency. Moreover, proteinuria at the time of discharge was observed to be lower when compared to that on admission (chi-square, p < 0.01, Supplementary Fig. 1).

### Subgroup analyses

In subgroup analysis, we assessed the association between proteinuria and outcome among patients with eGFR < 60 mL/min/1.73 m^2^ and those with eGFR ≥ 60 mL/min/1.73 m^2^. In the analysis using renal parameters before admission, proteinuria before admission was associated neither with oxygen administration nor developing pneumonia both among patients with eGFR < 60 mL/min/1.73 m^2^ and those with eGFR ≥ 60 mL/min/1.73 m^2^ (Tables 5 and 6). In the analysis using renal parameters on admission, proteinuria on admission was not independently associated with oxygen administration not among patients with eGFR < 60 mL/min/1.73 m^2^ but among those with eGFR ≥ 60 mL/min/1.73 m^2^ (OR 3.28; 95% CI 0.92–11.72, p = 0.07 and OR 4.86; 95% CI 1.22–19.38, p = 0.03, respectively, Tables 5). Conversely, proteinuria on admission was independently associated with developing pneumonia both among patients with eGFR < 60 mL/min/1.73 m^2^ and those with eGFR ≥ 60 mL/min/1.73 m^2^ (OR 6.45; 95% CI 1.78–23.35, p < 0.01 and OR 3.65; 95% CI 1.06–12.58, p = 0.04, respectively, Tables 6).

**Table 5:** Factors contributing to the requirement of oxygen administration in patients with proteinuria

Variable	Using renal parameters before admission						Using renal parameters on admission					
	eGFR < 60 mL/min/1.73 m^2^			eGFR ≥ 60 mL/min/1.73 m^2^			eGFR < 60 mL/min/1.73 m^2^			eGFR ≥ 60 mL/min/1.73 m^2^		
	Odds ratio	95% CI	p value	Odds ratio	95% CI	p value	Odds ratio	95% CI	p value	Odds ratio	95% CI	p value
Age [years]	1.03	0.97–1.09	0.29	1.05	1.02–1.09	< 0.01	1.06	0.99–1.13	0.10	1.05	1.00–1.10	0.05
Male gender	0.43	0.06–2.88	0.38	2.22	0.71–6.95	0.17	2.27	0.31–16.42	0.42	5.31	0.95–29.63	0.06
BMI [kg/m^2^]	1.06	0.94–1.18	0.34	1.01	0.90–1.12	0.92	1.10	0.96–1.26	0.18	1.05	0.91–1.22	0.49
Smokers	6.77	0.96–47.84	0.06	0.90	0.34–2.39	0.83	1.71	0.27–10.79	0.57	0.30	0.07–1.29	0.11
Diabetes	1.21	0.40–3.68	0.74	1.67	0.66–4.21	0.28	1.50	0.44–5.14	0.52	1.03	0.26–4.11	0.97
Cardiovascular disease	0.21	0.03–1.35	0.10	1.46	0.36–5.98	0.60	0.14	0.02–0.91	0.04	2.18	0.35–13.67	0.41
Lung disease	2.71	0.86–8.57	0.09	0.89	0.32–2.46	0.82	3.11	0.76–12.77	0.12	0.78	0.17–3.72	0.76
Proteinuria ≥ 1 +	0.70	0.21–2.34	0.57	1.62	0.46–5.68	0.45	3.28	0.92–11.72	0.07	4.86	1.22–19.38	0.03

*CI*, confidence interval; *BMI*, body mass index; *eGFR*, estimated glomerular filtration rate

**Table 6:** Factors contributing to the developing pneumonia in patients with proteinuria

Variable	Using renal parameters before admission						Using renal parameters on admission					
	eGFR < 60 mL/min/1.73 m^2^			eGFR ≥ 60 mL/min/1.73 m^2^			eGFR < 60 mL/min/1.73 m^2^			eGFR ≥ 60 mL/min/1.73 m^2^		
	Odds ratio	95% CI	p value	Odds ratio	95% CI	p value	Odds ratio	95% CI	p value	Odds ratio	95% CI	p value
Age [years]	1.02	0.97–1.08	0.36	1.01	0.99–1.04	0.27	1.00	0.94–1.05	0.88	1.02	0.99–1.06	0.21
Male gender	0.27	0.04–1.62	0.15	1.31	0.55–3.07	0.54	0.45	0.07–2.88	0.40	1.13	0.33–3.84	0.84
BMI [kg/m^2^]	1.04	0.93–1.15	0.53	1.02	0.94–1.11	0.63	1.02	0.90–1.17	0.75	1.07	0.95–1.21	0.24
Smokers	9.24	1.60–53.42	0.01	0.57	0.25–1.28	0.17	4.62	0.78–27.36	0.09	0.39	0.12–1.31	0.13
Diabetes	0.67	0.23–1.93	0.45	1.28	0.58–2.82	0.55	0.60	0.17–2.09	0.42	1.58	0.52–4.81	0.42
Cardiovascular disease	5.21	0.81–33.56	0.08	1.74	0.46–6.59	0.41	1.17	0.25–5.37	0.84	2.44	0.41–14.56	0.33
Lung disease	1.07	0.35–3.31	0.91	1.04	0.42–2.55	0.94	1.97	0.47–8.25	0.35	1.15	0.30–4.40	0.84
Proteinuria ≥ 1 +	1.15	0.37–3.55	0.81	1.00	0.32–3.11	1.00	6.45	1.78–23.35	< 0.01	3.65	1.06–12.58	0.04

## Discussion

This study showed that underlying proteinuria before admission was not associated with the requirement for oxygen administration and developing pneumonia. Nevertheless, proteinuria on admission after the onset of COVID-19 was associated with oxygen administration and developing pneumonia. Proteinuria on admission may reflect the intensity of renal manifestations because the percentage of patients with proteinuria on admission was significantly higher than those before admission.

According to our knowledge, this is the first study to look at a connection between underlying proteinuria and the severity of COVID-19. However, there are several studies of the association between underlying reduced eGFR and COVID-19 severity in non-dialysis-dependent CKD. For example, the largest study from the United Kingdom at the early phase of its epidemic reported that underlying reduced eGFR was associated with COVID-19-related deaths (adjusted hazard ratio 2.52 for patients with eGFR < 30 mL/min/1.73 m^2^ and 1.33 for patients with eGFR 30–60 mL/min/1.73 m^2^) [2]. A significant link between underlying reduced eGFR and COVID-19-related mortality was also demonstrated by other investigations [24–26]. Similarly, in our study, reduced eGFR before admission was associated with oxygen demand suggestive of severe COVID-19.

In-hospital mortality and the requirement for mechanical ventilators, both of which are often used as outcomes in previous relevant studies, were relatively rare in this study [2, 24–26]. This is probably due to recent advances in treatment, the widespread availability of vaccines, viral mutations, and the efforts of our staff. Living guidance for clinical management of COVID-19 by the World Health Organization describes “signs of pneumonia” or “oxygen saturation < 90%” as “severe”. Conversely, the severity classification of Japanese clinical guidance for COVID-19 defines “oxygen administration required” or “SpO_2_ ≤ 93%” as “severity of Moderate II,” and “shortness of breath and pneumonia findings” or “93% < SpO_2_ < 96%” as “severity of Moderate I.” Therefore, in this study, oxygen administration and developing pneumonia were included as outcomes related to COVID-19 severity [27].

This study also showed that the value of renal parameters significantly worsened soon after infection than before, which could be the direct and indirect effects of COVID-19. Although there are several studies on changes in eGFR before and after infection as those of prehospital AKI, no studies have investigated changes in proteinuria [26, 28]. Note that the degree of their worsening may be affected by the time from infection to diagnosis or hospitalization, which may vary depending on the burden of medical care in each country.

The next issue is the risk of proteinuria and reduced eGFR on admission. Proteinuria before admission was not associated with either oxygen administration or developing pneumonia, but proteinuria on admission was associated with both of them. We found that CRP and hematuria were associated with positive proteinuria in various inflammation-related parameters. We also found that some of these parameters were associated with severe clinical outcomes. These results suggest that proteinuria has a relationship with inflammation and the severity of COVID-19. Several reports have also shown an association between proteinuria on admission and COVID-19 severity [29–32]. Conversely, it is also known that there is an association between reduced eGFR on admission or prehospital AKI and COVID-19 severity [33–37]. Similarly, in this study, reduced eGFR on admission was more associated with oxygen demand than that before admission. Taken together, values of renal parameters on admission appear to be more associated with COVID-19 severity. We believe that the reason is that values on admission may already reflect the intensity of COVID-19 symptoms. Hence, the measurement of proteinuria, and eGFR on admission would be informative for risk stratification during hospitalization.

Intriguingly, proteinuria on admission appears to be more closely associated with COVID-19 severity than reduced eGFR. Moreover, vascular endothelial dysfunction, which may be the primary pathophysiology of proteinuria, is known to be associated with cytokine storm [12, 19, 20]. This fact could explain the strong association between proteinuria and respiratory symptoms, such as oxygen administration and developing pneumonia [38–40].

This research had several limitations. First off, because this research was conducted at a single facility in Japan, genetic background, national medical systems, and the features or circumstances of each hospital all have a significant impact on the outcomes [41, 42]. Second, despite the current recommendations for the use of quantitative measurement of albuminuria for accurate CKD diagnosis, semiquantitative measurements of proteinuria by dipstick test, which is less sensitive but is still widely used in everyday clinical practice because it is cheap, were employed in this study [43–45]. Third, in contrast to the description in the guidelines, CKD patients are only identified using a single measurement for proteinuria and a reduced eGFR without proof of chronicity, which could result in overdiagnosis [46, 47].

## Conclusions

There was no association between proteinuria before admission and COVID-19 severity. However, proteinuria on admission was associated with oxygen administration and developing pneumonia. The percentage of patients with proteinuria on admission was significantly higher than those before admission. Although there have been several reports that demonstrate the association between eGFR (or proteinuria) at diagnosis of COVID-19 and COVID-19 severity, these variables are also impacted by the intensity of COVID-19, and vice versa. Proteinuria and eGFR before the onset of COVID-19 should be used as explanatory factors to investigate the impact of underlying CKD per se on COVID-19 severity. We believe that it would be beneficial for future studies on proteinuria and CKD as well as risk classification in COVID-19.

### Supplementary Information

Below is the link to the electronic supplementary material.

Supplementary Methods: Cardiovascular disease was defined as heart failure, pulmonary edema, acute myocardial infarction, angina pectoris, aortic dissection, arrhythmia, endocarditis, and valvular disease. The term “lung disease” was used for interstitial pneumonia, asthma, pulmonary fibrosis, empyema, chronic obstructive pulmonary disease, sarcoidosis, tuberculosis, nontuberculous mycobacterial lung disease, hypersensitivity pneumonitis, eosinophilic granulomatosis with polyangiitis, pulmonary thromboembolism, bronchiectasis, and lung cancer (XLSX 13 kb)

Supplementary file2 (pptx 51 kb)

## Data Availability

The datasets used and analyzed during the current study are available from the corresponding author upon reasonable request.
