# Supplementary figures and images for: The relation between proteinuria and the severity of COVID-19
Source: Clin Exp Nephrol. 2023 Nov 14;28(3):235–44. doi: 10.1007/s10157-023-02428-9 (PMC10881620; doi:10.1007/s10157-023-02428-9)

## Slide 1
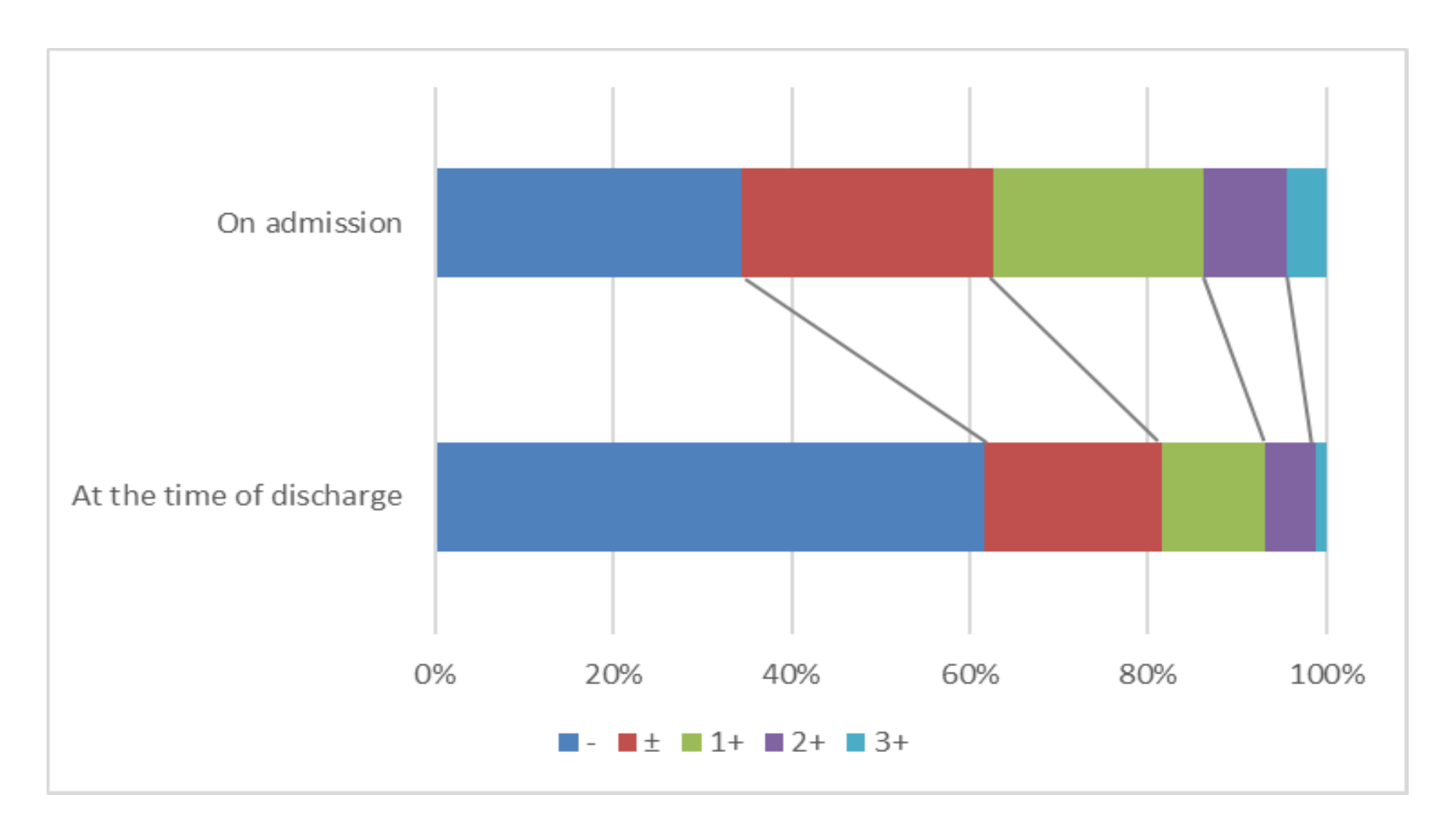

Supplement: Supplementary file 2 — Supplementary file2 (pptx 51 kb) [file 10157_2023_2428_MOESM2_ESM.pptx]
